# Small-scale health-related indicator acquisition using secondary data spatial interpolation

**DOI:** 10.1186/1476-072X-9-50

**Published:** 2010-10-13

**Authors:** Gang Meng, Jane Law, Mary E Thompson

**Affiliations:** 1 School of Planning, University of Waterloo, Waterloo, Ontario, N2L3G1, Canada; 2 Department of Health Studies and Gerontology, University of Waterloo, Waterloo, Ontario, N2L3G1, Canada; 3 Department of Statistics and Actuarial Science, University of Waterloo, Waterloo, Ontario, N2L3G1, Canada

## Abstract

**Background:**

Due to the lack of small-scale neighbourhood-level health related indicators, the analysis of social and spatial determinants of health often encounter difficulties in assessing the interrelations of neighbourhood and health. Although secondary data sources are now becoming increasingly available, they usually cannot be directly utilized for analysis in other than the designed study due to sampling issues. This paper aims to develop data handling and spatial interpolation procedures to obtain small area level variables using the Canadian Community Health Surveys (CCHS) data so that meaningful small-scale neighbourhood level health-related indicators can be obtained for community health research and health geographical analysis.

**Results:**

Through the analysis of spatial autocorrelation, cross validation comparison, and modeled effect comparison with census data, kriging is identified as the most appropriate spatial interpolation method for obtaining predicted values of CCHS variables at unknown locations. Based on the spatial structures of CCHS data, kriging parameters are suggested and potential small-area-level health-related indicators are derived. An empirical study is conducted to demonstrate the effective use of derived neighbourhood variables in spatial statistical modeling. Suggestions are also given on the accuracy, reliability and usage of the obtained small area level indicators, as well as further improvements of the interpolation procedures.

**Conclusions:**

CCHS variables are moderately spatially autocorrelated, making kriging a valid method for predicting values at unsampled locations. The derived variables are reliable but somewhat smoother, with smaller variations than the real values. As potential neighbourhood exposures in spatial statistical modeling, these variables are more suitable to be used for exploring potential associations than for testing the significance of these associations, especially for associations that are barely significant. Given the spatial dependency of current health-related risks, the developed procedures are expected to be useful for other similar health surveys to obtain small area level indicators.

## Background

Remarkable geographic variations in health have been displayed in all developed countries. Over the centuries, despite overall improvements in health and longevity, spatial and social disparities of the incidence and prevalence of chronic diseases have not lessened, even in a country with universal health care, such as Canada [1]. Moreover, along with the globalization of the world economy and the polarization of socio-economic classes, health gaps have even increased between the rich and the poor and between the socially included and excluded. As is increasingly being recognized, the interplay of spatial and social determinants has shaped the landscapes of health inequalities.

Contextual influences, such as neighbourhood characteristics, have become the focus of health planners and health geographers in addressing health inequality issues. A majority of such neighbourhood or small-area analyses focus on the aspects of socio-economic status, ethnicity, demography, and physical living conditions and their consequences on population health. This is largely due to the wide availability of census data, which can be aggregated at different spatial levels to obtain necessary indicators mentioned above. However, other important aspects of social determinants of health, such as psycho-social risk factors, health service provision, food supply, walkable and liveable communities, urban design features, and average community health conditions, have not been fully investigated at the neighbourhood level due to the difficulties in obtaining corresponding indicators. The lack of evidence for these kinds of social determinants has prevented public health interventions from addressing their potential risks to population health and contributions to health inequalities. Effective neighbourhood-level potential risk factors need to be identified and corresponding data need to be collected or derived at different spatial scales to make the analyses possible.

Nowadays, because of the awareness of the importance of health research, various kinds of health-related data are collected and maintained by health researchers and practitioners, including census data, health survey data, and administrative data, many of which are geo-referenced by either home addresses or postal codes of individual records. This increasing availability of geo-referenced health-related data provides more opportunities for geographical analyses to address social and environmental health problems. However, since many of the data are collected according to their designated primary purposes, they cannot be utilized directly in secondary data analyses due to sampling issues, especially when indicators are required at very small areal units. These data need to be effectively handled and interpolated before they can be used for geographical analyses to explore the relationships between environment and health.

One potential Canadian data source of community-health indicators is the Canadian Community Health Surveys (CCHS). The CCHS is a cross-sectional survey that collects information related to health status, health care utilization and health determinants for the Canadian population. It includes common content ranging from Alcohol Use, General Health, Health Care Utilization, and Exposure to Second-hand Smoke, through Physical Activities and Income; and optional content, such as Access to Health Care Services, Stressors, Depression, Food Security, Health Care System Satisfaction, Home Safety, Satisfaction with Life, Self-Esteem, Social Support, Voluntary Organizations, and Work Stress. These variables cover a wide range of health-related risks and may have potential uses for the analyses of social and environmental impacts on population health.

However, CCHS surveys are designed and collected for community health research at the health region level, which is much larger than the neighbourhood level required for small-area analysis. CCHS samples are allocated proportionally to the square root of the population in each health region. In Ontario, 50% of the sample units are selected from an area frame and 50% from a list frame of telephone numbers. The area frame uses a stratified two-stage design. The first stage of sampling consists of selecting randomly smaller geographic areas, or clusters, from within each stratum. The second stage of sampling consists of selecting randomly dwellings from within each selected cluster. The list frame select telephone numbers using a random sampling process in each health region. Based on this sample design, samples are not uniformly distributed among smaller areal units, such as census Dissemination Areas (DAs). Sample sizes are much larger in urban areas than in rural areas. At each cycle (specifically, 1.1, 2.1 and 3.1), a certain number of DAs in rural areas do not contain even one sample point. Therefore, it is not feasible to aggregate directly individual-level variables to obtain small area level indicators.

The objectives of this paper are to identify appropriate procedures and interpolation methods to generate small-area-level health-related risk factors for community health analyses using Canadian Community Health Survey (CCHS) data, and to incorporate the derived variables in hierarchical modeling to identify the possible impacts of community-level risks on population health.

## Methods

### Theoretical bases of spatial dependency

Determinants of health operate at multiple levels, including intrapersonal, interpersonal, organizational, community and society levels [2]. It is the interplay of determinants at all levels that affects an individual's health. Geographical analyses focus on risk factors at the community level, especially the geographical aspects of communities, namely neighbourhoods, and their interrelations with lower-level risks in influencing health outcomes. Neighbourhoods refer to spatial areas that represent individuals' places of residence and the surrounding environments where people live and interact with each other. Influenced by personal choices or external forces, people with common socio-economic status, ethnicity or demographic characteristics tend to live close to each other within a city. They consider certain surrounding areas as their neighbourhoods or their communities that provide safe places and serve as important sources of support and sociability [3].

Although it is argued that individuals are isolated in modern industrialized urban life and local neighbourhoods lack social ties to provide solidarity and assistance [4], neighbourhoods are still functioning as containers or constraints that limit local residents' access to service facilities, resources, education and job opportunities, constrain their interactions and even the selection of marriage partners [5], and affect and regulate their behaviours or beliefs through common norms. Within their neighbourhood, residents are continually involved in neighbourhood affairs, activities, and interactions through community-level organizations and day-to-day communications. In addition, potential environmental risks, such as air pollution, may also affect the health conditions of residents living in nearby neighbourhoods. Due to these impacts, homogeneities may be found within neighbourhoods or surrounding areas, in terms of living conditions, socio-economic status, health-related behaviours, psycho-social status, and general health. These geographically associated neighbourhood characteristics provide directions for public health interventions to address population health issues.

Neighbourhood characteristics (or collectives) are often associated with individual-level characteristics (or members), but are not necessarily based on individual characteristics [6]. Some collective characteristics can be obtained by aggregating information from individual members, such as the average income of an area. Some are based on the relationships between members, such as the social network of a neighbourhood. Others are the characteristics of the collective itself, such as health care provisions or policies. However, the collective properties at the neighbourhood level are sometimes difficult to obtain, or obtain directly. Samples for most health surveys are individual-based, rather than collectivity-based. Some neighbourhood properties are multidimensional and hard to measure, such as social capital, which includes aspects of social interactions, social network, trust, and mutual benefits. Some other properties may be affected by spatial and social distances and therefore may be hard to divide among neighbourhoods, such as health provisions.

In the absence of directly measured neighbourhood-level variables or indicators, an alternative method to obtain health-related neighbourhood properties is through the aggregation of individual-level characteristics in surveys so that common characteristics may be found to represent collective properties. For example, although social capital is a collective property of neighbourhoods, it may be represented by local residents' average sense of belonging to local communities.

### Spatial interpolation

The spatial dependencies or homogeneities of neighbourhood characteristics legitimate the use of spatial interpolation methods to obtain small-area variables. Based on spatial dependencies, spatial interpolation methods predict values for specified spatial locations using a limited number of sample data points at nearby locations. These methods can be grouped into deterministic and stochastic methods.

Deterministic methods interpolate values based on either the extent of similarity, such as the Inverse Distance Weighting (IDW) method, or the degree of smoothing as in the use of radial basis functions, such as spline and multiquadric functions. The methods can be either global or local, and can be exact (such as IDW, and spline) or inexact (such as polynomial interpolators). On the other hand, stochastic interpolation techniques, such as kriging, quantify the spatial autocorrelation among sample points based on the entire dataset within the study area, and account for the spatial configuration of the sample points around the prediction locations for interpolation. Although kriging is sometimes described as a local interpolation method since technically more weights are given to neighbouring observations, stochastic interpolation methods are by nature global interpolation techniques. The discussion and analysis in this paper focus on two commonly adapted interpolators, namely IDW and kriging.

A widely used deterministic interpolation method is Inverse Distance Weighting (IDW). It is a local exact interpolator that interpolates values based only on the surrounding measured values of the interpolating location and functions of the inverse distances between the interpolating location and locations of the surrounding sample. Shepard's IDW interpolation method [7] seeks to find an interpolated value *z *at a given point ***x ***based on samples *z*_*i *_*= z(x*_*i*_*) *for *i = 0,1, ..., n*. This method can be written as:

$$z(x)=\sum_{i=0}^{n}\frac{w_{i}(x)}{\sum_{i=0}^{n}w_{i}(x)}z_{i}$$

where $w_{i}(x)=\frac{1}{d{\left(x,x_{i}\right)}^{p}}$ is the weighting function denoted by the inverse Euclidean distance between the interpolating point *x *and the neighbouring data point *x*_*i*_, raised to the power *p*.

Modifications to the weighting function for interpolation are also suggested. One modification is Liszka's method [8], which can be expressed as $w_{i}(x)=\frac{1}{{\left(d(x,x_{i})+\delta^{2}\right)}^{p}}$, where *δ*^*2 *^is a constant dependent on the measurement error to control the smoothness of interpolation. The interpolated surface becomes smoother with the increase of the tuning parameter *δ*^*2*^. The other modification is the Lukaszyk-Karmowski metric, which is defined by $w_{i}(x)=\frac{1}{D_{RD}{\left(x,x_{i}\right)}^{p}}$, where *D*_*RD*_*(x,x*_*i*_*) *is the probability metric of random values *x *and *x*_*i *_assuming they have uniform and Dirac delta distribution respectively [9]. This approach is physically based, allowing the real uncertainty in the location of the sample point to be considered.

The IDW interpolation depends on the selection of the power value, *p*, and the neighbourhood search strategy. It is an exact interpolator, where the interpolated surface goes through the sample points and maximum/minimum values in the surface only occur at sample points. IDW assumes that the spatial process at the interpolating locations is being driven by the local variation only, which can be captured through the surrounding sample points. The output values therefore are sensitive to local spatial clustering and the presence of outliers. Health related variables that are determined by local sources of environmental risks, such as pollution from local industries, may fall into this category.

On the other hand, stochastic methods, such as kriging [10,11], interpolate values not only based on the surrounding data values, but also based on the overall autocorrelation calculated by applying statistical models to all the known data points. Because of this, not only do stochastic methods have the capability of producing a prediction surface, but they also provide some measure of the certainty or accuracy of the predictions. The kriging method can be similarly constructed as:

$$\hat{z}(x)=\sum_{i=0}^{n}w_{i}(x)z(x_{i})$$

where the interpolated value $\hat{z}$ at point *x *is the weighted sum of the neighbouring observed values *z*_*i *_*= z(x*_*i*_*) *with weights *w*_*i*_*(x), i = 1, ..., n*, chosen such that the kriging variance is minimized, subject to the unbiasedness condition:

$$E[\hat{z}(x)-z(x)]=\sum_{i=0}^{n}w_{i}(x)\mu (x_{i})-\mu (x)=0$$

where *μ(x) = E[z(x)] *is the expected value of *z(x)*. This means that the interpolated value at a given location is the sum of two components: an unknown underlying surface defined by *μ(x)*, plus some additional noise.

Based on different assumptions on *μ(x) *and the unbiasedness condition for calculating the weights, different types of kriging apply, including simple kriging, ordinary kriging, universal kriging, IRFk-kriging, indicator kriging, disjunctive kriging, lognormal kriging, and co-kriging.

The most commonly used type of kriging is ordinary kriging. It assumes a wide sense stationary process with a constant but unknown mean, *μ(x) = μ*, over the study region. Similar with the concepts of the IDW interpolation, the weights, *w*_*i*_*(x)*, decline as distances between the points at which the surface is being estimated and the locations of the data points increase. The difference is that, instead of using the local inverse distance function, the kriging method uses the globally calculated semi-variogram [12] to calculate the weights.

The semi-variogram is a function describing the degree of spatial dependence of a variable *z(x)*. It is usually represented as a graph that models the autocorrelation among sample locations to identify how values vary with distance of separation. If there are enough observations and there is no directional effect, a semi-variogram can be empirically estimated by a semi-variogram cloud, *γ(h)*, which is defined as

$$r(h)=\frac{1}{2}\frac{1}{n_{h}}\sum_{i=0}^{n_{h}}{\left(z(x_{i}+h)-z(x_{i})\right)}^{2}$$

where h is lag distance, *n*_*h *_is the number of paired observations at the distance h, and z is the observed value at a particular location. The semivariance at lag distance h, *γ(h)*, is half the variance of the differences *z*(*x*_*i *_+ *h*) - *z*(*x*_*i*_), which is equivalent to the whole variance of z-values at distance *h *[13]. If a directional effect exists, the selection of paired observations to construct the semi-variogram is determined not only by their Euclidian distances but also by their spatial directions.

The semi-variogram can then be plotted against a number of lags. To ensure validity for kriging, the *γ(h) *are often approximated by model functions, such as exponential, spherical, and Gaussian functions. The parameters that affect a typical fitted semi-variogram function are nugget, range, and sill.

An appropriately fitted semi-variogram should be able to reveal the real scale-dependent spatial correlation. In addition to function and parameter selections, the choice of lag sizes and number of lags also affects the fit of the selected function to the empirical semi-variogram. Excessively large lag sizes may mask short-range autocorrelation while excessively small lag sizes may be associated with small sample sizes within each lag to achieve representative averages of *γ(h)*. The number of lags should also be selected carefully so that the range of the semi-variogram function is less than the maximum estimating distance, obtained by multiplying the lag size by the number of lags. If the range of the fitted semi-variogram model is small relative to the extent of the empirical semi-variogram, the lag size or number of lags can be reasonably decreased to reduce estimating time without sacrificing general accuracy.

Once the semi-variogram model is fit, it can then be used to calculate the weights, *w*_*i*_*(x)*, for kriging. Since kriging is estimated using neighbourhood points of the predicting location, the selection of neighbourhood structure or number of neighbourhood points also has impact on the kriging results.

The assumption of ordinary kriging that the study region has a wide sense stationary process with a constant mean is largely consistent with current regional-level health status in developed countries, such as Canada. Due to the already advanced medical care and the fact that chronic diseases are the leading cause of mortality and morbidity, health status and life expectancies remain relatively stable in most developed societies. Neighbourhood differences in health-related status may be seen as spatially autocorrelated random effects over or below the average health status. It may therefore appropriate to use ordinary kriging for interpolating most of the CCHS variables. However, as mentioned earlier, for variables that are potentially influenced by one or several common sources of risks, a globally applicable semi-variance function of spatial dependency may not exist. In this case, local estimators, such as IDW, may be more appropriate to use. Values at unknown locations can be estimated locally using only nearby samples and distance decay factions. In addition, if a recognizable spatial trend exists, for example, a distance decayed environmental influence on health from a common source of risk or potential rural-urban differences for neighbourhood incomes, other kriging methods, such as universal kriging, may be applied to model the trend as a polynomial surface and perform kriging on the residuals. Thus, the selection of interpolation methods and parameters are determined by the spatial processes of specific variables. In this paper, the effects of the IDW and kriging interpolation methods and different choices of parameters are compared to seek well-performing solutions for CCHS data interpolation.

Using a cross validation method, which takes a data point out of the fitting and then predicts its value and compares the prediction to its actual value, the accuracy and unbiasedness of different interpolation methods or different selection of parameters of the same method can be compared. In particular, the root-mean-square prediction error, which quantifies the root mean square difference between predicted and measured values at sample locations, can be compared between different models as a way of choosing one model over another or adjusting parameter values. Since neighbourhood-level socio-economic status (such as income, education, and occupation) can be easily obtained from census data, it is also feasible to compare directly the interpolated CCHS socio-economic status variables, such as household income, with the corresponding variables from the Census to demonstrate the accuracy of the spatial interpolation using CCHS data or other potential data sources.

Once an effective interpolation method and its corresponding parameters are determined after comparison, a two-step procedure is taken to obtain desired neighbourhood-level variables. First, a smoothed surface with interpolated values at regular grid points over the study area is created using the selected method and parameters. Second, based on defined neighbourhood-level or small-area level boundaries, the interpolated surface is aggregated to obtain values for each neighbourhood or small area unit. Neighbourhoods often do not have clearly defined boundaries. Different people may perceive different geographical division of neighbourhoods based on their own experience. Neighbourhood boundaries are also changing over time based on changing social and spatial processes. In reality, most definitions of neighbourhood use proxy bureaucratic boundaries, such as census tracts or census dissemination areas, which are also changing from census to census. The inconsistency of neighbourhood boundaries over time is an obstacle, in health studies, to comparison, trend analysis, and prediction. The advantage of this two-step approach is that the interpolated surface in the first step allows data to be aggregated to any desired small area level units. Once neighbourhood boundaries are determined based on specific studies, data obtained at different periods of time can be interpolated and aggregated to the same areal units using the proposed steps, making temporal or longitudinal studies possible.

A case study is conducted in the city of Windsor and Essex County in Ontario, Canada to illustrate the procedures of determining the appropriate interpolation method and data handling processes, and to demonstrate the use of the interpolated results for analyzing neighbourhood impacts on health, specifically on low birth weight (LBW).

## Empirical study

The study area is chosen in the health region of Essex County and the City of Windsor in Ontario, Canada, because of the availability of individual data on birth outcomes and associated risks between 2000 and 2008 in this region. This makes it possible to construct multi-level statistical models to analyze the social and spatial determinants of adverse birth outcomes.

As with most health outcomes, factors influencing LBW births operate at multi-levels. Most previous research has studied risk factors at the personal and interpersonal level. Various risk factors can be identified, including biological and psychological factors, maternal demographic and anthropometric factors, genetic factors, maternal medical factors, maternal trauma, nutritional factors, infections, multiple births, stress, lifestyles, family violence, family socioeconomic factors, and marriage status [14,15]. At the macro-level, health inequality theories [16-19] suggest that LBW births are potentially associated with various social and environmental risks, including cultural and behavioural risks, material and social structural risks and neighbourhood-level psycho-social risks. Some of these risks, such as work conditions [20], air pollution [21], neighbourhoods of low socio-economic status [22], ethnic composition [23], and neighbourhood-level stress and adaptation [24], are studied and associations are established. However, the impacts of some other potential neighbourhood-level risk factors, such as social capital, community organization, public facilities, health service provision, and healthy food provisions, on LBW are not studied or not fully studied. A plausible obstacle may be the lack of related data at small area level.

CCHS data are therefore used to obtain small-area-level health-related variables for this analysis. The individual-level variables among different cycles of CCHS data are mostly consistent and samples for each cycle are randomly selected. This provides the possibility of combining samples from different cycles to obtain appropriate sample sizes for aggregation or spatial interpolation. Once samples of relevant and consistent variables are combined and extracted from different cycle surveys, different spatial interpolation methods can be applied and compared to obtain estimates at unknown locations (usually at regulator grid pints) over the study region. Neighbourhood level variables at different spatial scales can then be obtained by aggregating the interpolated grid points.

Fourteen variables that may be proxies of environmental characteristics and have potential influences on LBW births are extracted from the three cycle (1.1, 2.1 and 3.1) CCHS data. Assuming the existence of potential environmental impacts, variables such as Self-Perceived Health (SPH) and Chronic Health Conditions (CHC) may represent environmental health conditions at the neighbourhood level. Self-Perceived Unmet Healthcare need (SPUH) may represent health service provisions. Self-Perceived Stress (SPS), Sense of Belonging to local Communities (SBC), and Emotional Unhappiness (EU) are potentially associated with neighbourhood-level social capital. Food Insecurity (FI) and Insufficient Vegetable Intake (IV) may indicate food provision level. Neighbourhood-level Daily Smoking (DS) and Smoking Inside Home (SIH) may represent environmental smoking. Physical Inactivity (PI) may be a result of poor community design or the lack of public facilities. Regular Drinking (RD) and Heavy Drinking (HD) at the neighbourhood level may also represent social drinking and potentially be related to social capital.

Although Household Income (HINC) can be easily obtained from other data sources, it is also extracted from the CCHS data so that it can be compared with the readily available DA-level Census 2006 data to assess the accuracy of the interpolation results. Since data from the latest three censuses show that there is very little difference over the years for the median household incomes in the study area ($49,018 in 1996, $52,680 in 2001, and $50,884 in 2006), it is not necessary to standardize or adjust them by years. The raw household incomes in 1000$ units in the three cycles of CCHS data and the 2006 Census were extracted and used directly.

By combining three cycles (1.1, 2.1, and 3.1) of CCHS data, 4095 samples were extracted over 648 Census 2006 DAs in the study region. Using the corresponding postal codes of these samples, 4086 samples were able to be geocoded by the postal code conversion file (PCCF+), and their spatial locations were obtained. Geocoding by postal code using the PCCF+ files is currently the best available method in Canada. Using this method, if one postal code area overlaps with several census units, such as DAs, samples that have the same postal code will be randomly assigned to a spatial location within one of these units proportional to the distribution of population within that postal code. This partially avoids samples with the same postal code being geocoded to one location.

However, samples with the same postal codes may still be geocoded to the same spatial location, especially when they are associated with only one census unit. Although it is possible to assign these samples randomly within their corresponding postal code area, they are assigned to the same representative point of the associated census unit using PCCF+[25]. The representative point is centrally located along a street segment for block-faces, and centrally located or population and dwelling weighted for dissemination blocks (DBs), dissemination areas (DAs), census subdivisions (CSDs), urban areas (UAs) and designated places (DPLs). Topology checks are also applied to ensure that the points fall within the appropriate geographic area. The representation points are therefore appropriate to be assigned to corresponding postal codes.

While randomly assigning locations within a postal code area may help to reduce the chances of getting false peak values in spatial analysis, it does not improve the accuracy of geocoding and consequently the construction of the semi-variogram, which may not be correctly constructed at small distances. It is therefore deemed better to assign samples to their most possible locations. This may result in a large nugget value of the semi-variogram comparing to the true distribution, but will have minimal influence on the semivariance-distance relations. In addition, to calculate the semi-variogram models using all geocoded samples, samples at the same locations were randomly moved a tiny offset (less than 1 metre) from their geocoded locations.

**Figure 1:**
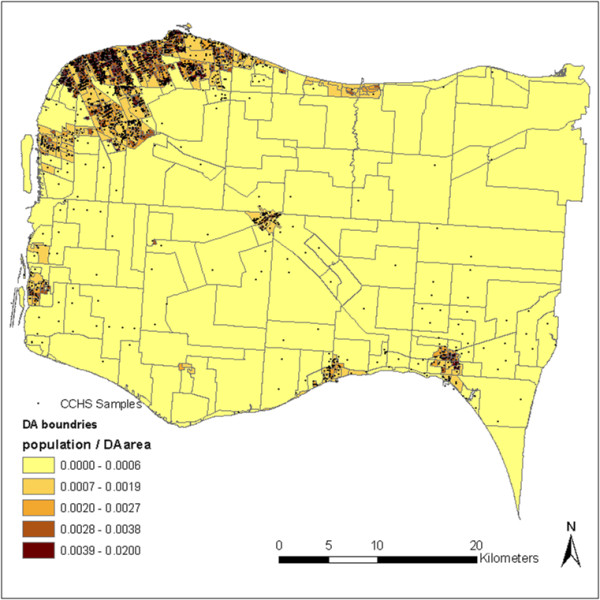
**Spatial distribution of CCHS samples among DA units in the study region**.

Figure 1 shows the spatial distribution of the geocoded CCHS samples. It can be seen that the sample points are mostly concentrated in cities and towns, where high population density can be observed. There is lack of sample points in some DAs, especially for rural areas (due to smaller population sizes); some of them have only a few sample points, or have no sample points at all. They therefore cannot be directly aggregated to derive DA-level variables. Certain spatial interpolation methods need to be employed before data can be aggregated to neighbourhood-levels. Rural areas are more subject to sampling issues due to the lack of sample points. However, the fact that rural areas are more homogeneous than urban areas in terms of socio-economic and other health-related status makes it legitimate for spatial interpolation to estimate variable values at unknown locations in these areas.

### Spatial autocorrelation among CCHS variables

Spatial interpolation methods are based on the assumption of spatial dependency. It is necessary to assess spatial autocorrelation before spatial interpolation is used. The Global Moran's I [26] method was used to assess the spatial dependency among CCHS variables. Given a set of sample locations and an associated attribute, Moran's I evaluates whether the pattern expressed is clustered, dispersed, or random. In general, a Moran's I index value near 1.0 indicates perfect clustering while an index value near -1.0 indicates perfect dispersion. The provided p-value also indicates whether or not the calculated index values are statistically significant, or in other words, whether or not the observed autocorrelations indicate truly non-zero dependence, or are just caused by chance.

An Inverse Distance Squared weighting scheme is applied to the obtained sample points to calculate the Moran's I values for selected CCHS variables. The neighbourhood search threshold is 4652 m, which is the largest distance between two neighbouring samples. The selection of this threshold value ensures that all sample points are used for calculating the spatial dependency in the study area. The resultant Moran's I values are relatively stable compared to those calculated from smaller threshold values, increasing slightly without changes in statistical significance. Table 1 lists the results, showing that most of the CCHS variables are spatially auto-correlated. Although the autocorrelations are moderate, they do show statistical significance. The existence of these spatial dependencies for most of the CCHS variables supports the use of spatial interpolation methods for estimating values at unknown locations. Several variables, including SPS, SIH, and HD do not show significant spatial dependencies globally. The use of spatial interpolation methods on these variables is questionable.

**Table 1:** Moran's I results for selected CCHS variables (Bold numbers represent statistically significant results at the 5% level)

CCHS variable	Moran's I Index	Variance	Z Score	p-value
**Household income (HINC)**	0.118910	0.000160	9.417029	**0.000000**

**Self-perceived health (SPH)**	0.101968	0.000161	8.064824	**0.000000**

**Chronic health conditions (CHC)**	0.030214	0.000161	2.402769	**0.016271**

**Self-perceived unmet health need (SPUH)**	0.005309	0.000171	0.425011	0.670829

**Self-perceived stress (SPS)**	-0.007969	0.000080	-0.862681	0.388313

**Sense of not belonging to local communities (SBC)**	0.024774	0.000161	1.9737	**0.0484**

**Emotional unhappiness (EU)**	0.063332	0.000079	7.149267	**0.000000**

**Food insecurity (FI)**	0.098504	0.000160	7.795798	**0.000000**

**Insufficient vegetable intakes (IV)**	0.030750	0.000161	2.444921	**0.014488**

**Daily smoking (DS)**	0.026619	0.000160	2.1218	**0.0339**

**Smoking inside home (SIH)**	-0.008169	0.000160	-0.6255	0.5316

**Physical inactiveness (PI)**	0.029967	0.000161	2.383148	**0.017165**

**Regular drinking (RD)**	0.033441	0.000161	2.657181	**0.007880**

**Hard drinking (HD)**	0.031225	0.000181	2.336443	**0.019468**

We examined how IDW and kriging methods and corresponding parameters may affect the interpolated results. The spatial interpolation tools in the Geostatistical extension in ESRI ArcGIS were used to conduct the analysis.

### Spatial interpolation method comparison and parameter selection

As mentioned earlier, the confirmed CCHS spatial dependence may be due to a process that applies universally across the study region, for example, distance impacting on the interaction and communication of local residents. In this situation, the kriging method may be a better choice for spatial interpolation. On the other hand, the spatial clustering may be a result of local constraints, such as the clustering of housing conditions caused by local zoning regulations. In this case, IDW may be a better choice. We examine both of the kriging and IDW methods using the household income variable in CCHS data and use the same variable obtained from the Census 2006 as the standard to compare the effectiveness of these methods.

**Figure 2:**
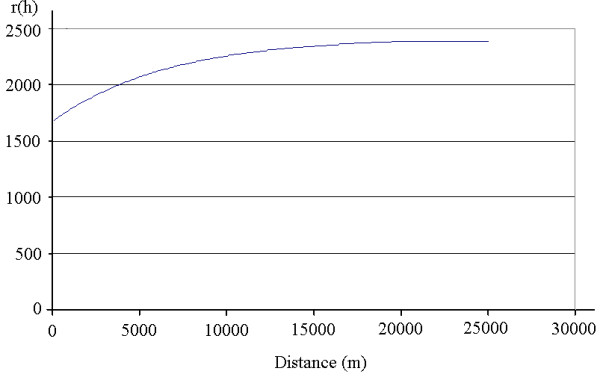
**The best fitted semi-variogram function for household income (in 1000$ units)**.

In addition to the IDW method, both universal kriging and ordinary kriging were applied to the household income variable to compare and assess the existence of potential spatial trends. However, the universal kriging results were no better than the ordinary kriging results and no recognizable spatial trend could be identified. This shows that the use of ordinary kriging assumptions on CCHS variables, discussed earlier, is warranted. Therefore, only selected selected cross-validation results using IDW and ordinary kriging with different parameters for interpolating the household income variable are listed in Table 2. It can be observed in this table that kriging methods perform better than IDW methods in terms of the root-mean-square prediction errors. This suggests the existence of underlying universally applied rules of spatial dependence. By adjusting the kriging parameters to obtain the minimum root-mean-square prediction error, the fitted semi-variogram function for the household income variable is produced in Figure 2.

**Table 2:** Cross-validation comparison between models and parameters

Spatial interpolation method and parameters	Mean prediction error	Root-Mean-Square	Average Standard Error	Mean Standardized	Root-Mean-Square Standardized
Ordinary kriging Exponential function lag: 200, number lags:100	-230.9	45360	41860	-0.005294	1.083

Ordinary kriging Exponential function lag: 100, number lags:100	-280.5	45360	43190	-0.00632	1.05

Ordinary kriging Exponential function lag: 200, number lags:50	-279.2	45360	43160	-0.006293	1.051

Ordinary kriging Spherical function lag: 200, number lags:100	-295.9	45400	42580	-0.006802	1.066

IDW with power 2	-906.4	50680			

IDW with power 1	-1060	48110			

The semi-variogram shows that the spatial dependency is largest at a small distance. The variogram values gradually increase with the increase of distance and reach the sill at about 20,000 metres. To ensure that short-range autocorrelation is not masked, a small lag size (200 m) was selected in this situation so that the fitted function may better represent the real distance-dependent spatial autocorrelation. 100 lags were then selected accordingly to cover the range of the semi-variogram. Due to small ranges for most of the CCHS variables, spatial dependencies only exist at small spatial distances. It is therefore better to use small lag sizes and a large number of lags to cover the ranges of the semi-variogram functions. Depending on the spatial structures of different CCHS variables, lag sizes between 50 m and 200 m were selected for the interpolation of variables of interest. The exponential and spherical functions show similarly good performance on the fit of the empirical semi-variogram. The selection of one over another is determined by comparing their corresponding root-mean-square prediction errors. In the case of household income interpolation, as shown in Table 2, the exponential function fits a little better than the spherical function and produces a relatively smaller root-mean-square error.

The selection of neighbourhoods is also affected by the small ranges of the semi-variogram. A small range in the semi-variogram means that spatial dependencies only exist at small distances. Therefore, selecting a large number of neighbouring samples over a large distance may not help to improve interpolation accuracy, but may produce an overly smoothed surface, which is not what we expect for values at small-area level. A suitable approach is to use samples from the same neighbourhood or surrounding neighbourhoods for interpolation so that local characteristics can be better preserved. Since some CCHS variables, such as SPH and CHC, may be affected by common risk sources, the spatial distribution of these variables may be potentially directional. Tests were therefore carried out in this paper comparing different neighbourhood structures using ellipse and circle shaped search ranges and dividing neighbourhoods into sectors. No directional effect was found and a fixed set of 50 nearest neighbours based only on Euclidean distance was finally chosen to make sure enough sample points are used for interpolation at each unknown location, and no unnecessary long-distance sample points are involved, so that local variations can be distinguished with a reasonable number of sample points.

**Figure 3:**
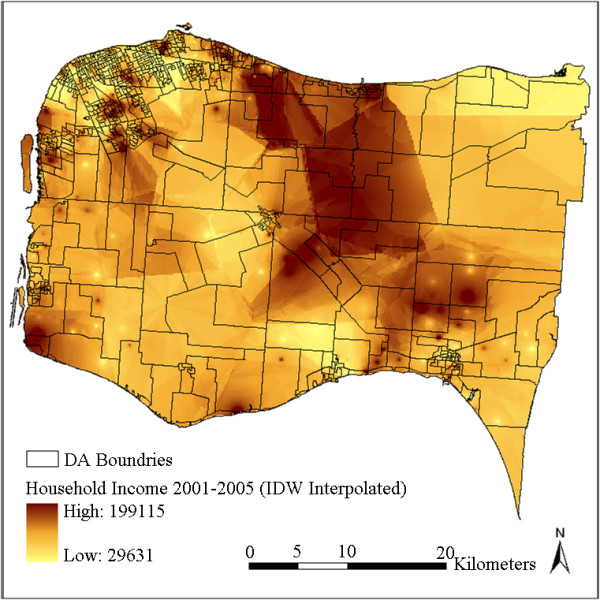
**Household income distribution interpolated using IDW with power 1**.

**Figure 4:**
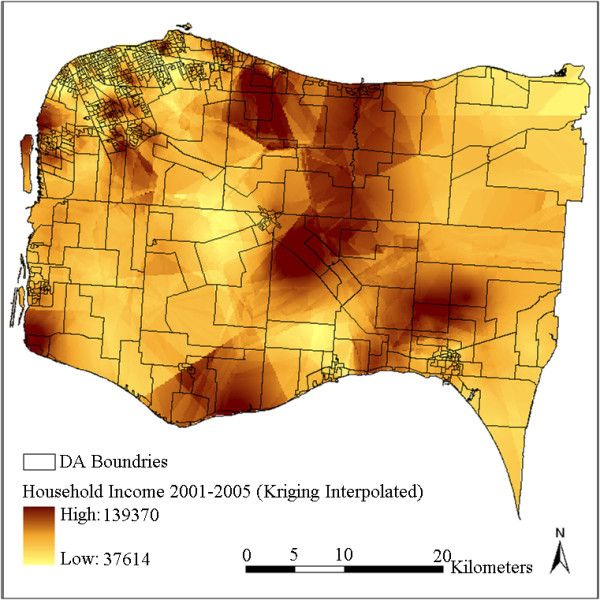
**Household income distribution interpolated using kriging**.

**Figure 5:**
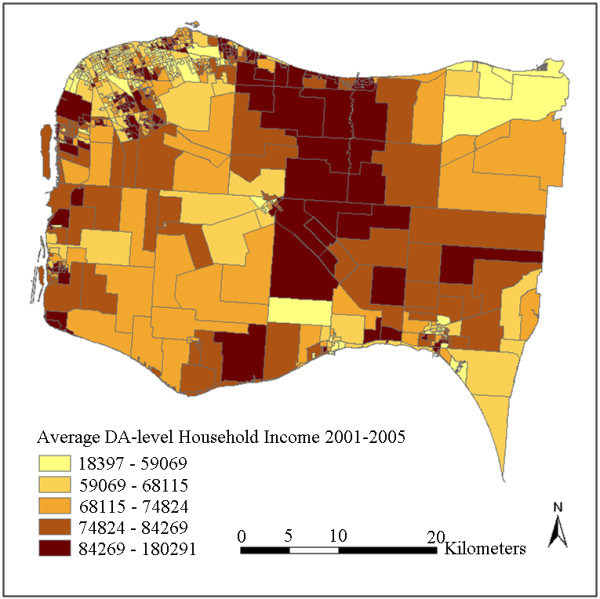
**Aggregated DA-level household income by IDW interpolation result**.

**Figure 6:**
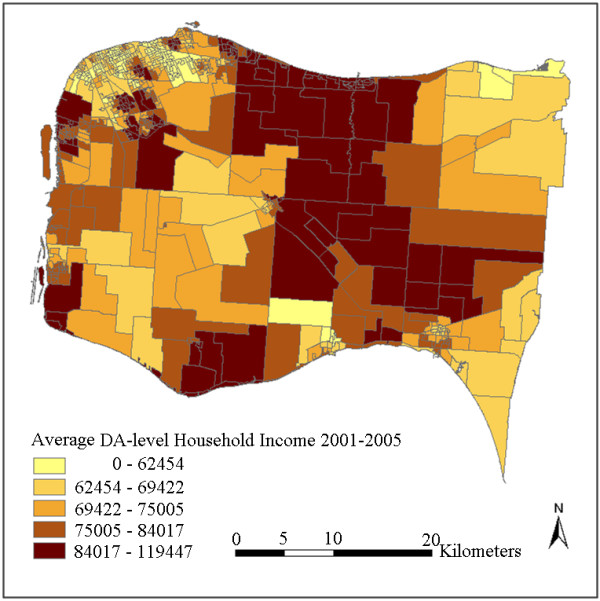
**Aggregated DA-level household income by kriging interpolation result**.

The interpolated surfaces using IDW interpolation with power 1 and using kriging with ideal parameters are plotted in Figure 3 and Figure 4. To further generate DA-level variables, these two surfaces were then aggregated by averaging the cell values in each DA respectively. The results are plotted in Figure 5 and Figure 6. It can be observed by comparing Figure 3 with Figure 4 (or comparing Figure 5 with Figure 6) that kriging interpolation results in a relatively smoother surface than the IDW method due to its use of the universally applied semi-variogram for weighting. The IDW interpolation, on the other hand, is subject to local spatial clustering. Hot spots of high interpolated values can be observed from place to place in Figure 3 due to high values or outliers in these locations.

**Figure 7:**
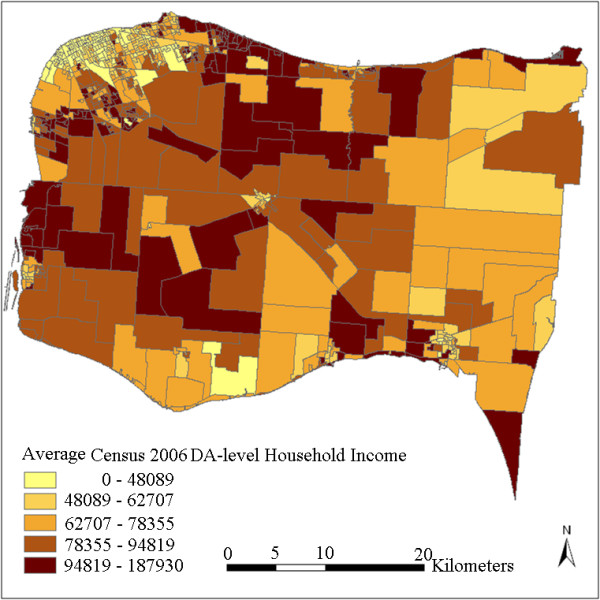
**Census 2006 DA-level household income**.

**Figure 8:**
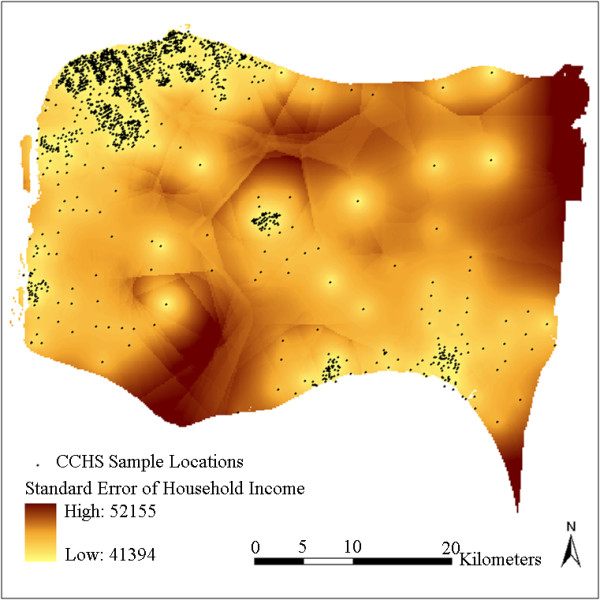
**kriging standard errors for the household income variable**.

To demonstrate the effectiveness of the interpolated results on the representation of the real DA-level status, DA-level household income values are extracted from the 2006 Census (Figure 7) for comparison. The standard errors produced by the kriging interpolation are also presented in Figure 8 to show the correctness and reliability of the interpolated results. A standard error is the standard deviation of the difference between the predicted and true values of a predicting location. It quantifies the uncertainty of the prediction for the location.

Although the CCHS samples were collected between 2001 and 2005 and the status of some DAs may have changed after that, the overall neighbourhood status of the study region has remained relatively constant. Figure 7 shows quite similar overall spatial patterns as Figure 5 and Figure 6, in that household incomes are lowest in cities and towns and higher in some sectors of suburb and rural areas. Differences can also be observed in that the census data shows a more random pattern and some of the values are not matched with the interpolated results, especially in some rural areas with less population. For example, some DAs at the upper-right and lower-right corners show inconsistent values. This can be partly explained by the larger standard errors due to lack of samples at these locations (Figure 8). This may also be due to some actual changes at the local area level, or data bias between the surveys and the census. Figure 7 shows that the actual spatial distribution of household income is determined by both global forces and local variance. The mean-squared-differences were also calculated between the kriging result and the census data, and between IDW results and the census data. kriging still shows a slightly better performance than IDW. This is consistent with the results presented in Table 2. Nevertheless, the overall gradients and patterns, and the relative status remain consistent between the interpolated results and the Census 2006 data.

Using similar procedures to those described above, other variables of interest described in Table 1 were also interpolated and aggregated to DA-levels to obtain neighbourhood-level indicators. These indicators were then used as explanatory variables in statistical modeling to assess the neighbourhood determinants of health, specifically on low birth weight.

### Statistical modeling using derived neighbourhood characteristics

Using the derived neighbourhood-level CCHS variables, multi-level models were constructed to assess the association between LBW and different neighbourhood-level risks. The models were written as

$$\begin{array}{l} LBW∼binary\left(p_{ij}\right)\\ \text{Level 1 }\left(\text{individual}\right):\\ logit\left(p_{ij}\right)=\beta_{0j}+\beta_{1}AGE19_{ij}+\beta_{2}AGE36_{ij}+\beta_{3}FEMALE_{ij}\\ \;\;\;\;+\beta_{4}MLTIBIRTH_{ij}+\left(\beta_{5}PRETERMBIRTH_{ij}\right)\\ \text{Level 2}\left(\text{neighbourhood}\right):\\ \beta_{0j}=\gamma_{00}+\gamma_{01}NB\_RISK_{j}+u_{0j} \end{array}$$

where *p*_*ij *_is the probability of having LBW for individual *i *in areal unit *j*. The coefficients *β*_*1*, _*β*_*2*_*,... β*_*m *_are parameters of individual-level risks. The function *logit(p*_*ij*_*) = log (p*_*ij *_*/(1- p*_*ij*_)), is a link function, which connects the linear predictors to the mean of the outcome variable (LBW), not directly to the outcome variable itself, so that the outcome variable can take on a non-normal form (binary distribution) to accommodate its non-continuity. The variable *β*_*0j *_is a random intercept at level 1, which is a function of the level 2 intercept *γ*_*00 *_and random effect *u*_*0j*_. *AGE19 *is an indicator for a teenage mother. *AGE36 *is an indicator for a mother of advanced age. *FEMALE *is an indicator for a female baby. *MLTIBIRTH *is an indicator for multiple births. *PRETERMBIRTH *is an indicator for preterm birth. By controlling mother's age, baby's sex, multiple births, and preterm births at the individual level, the birth outcome may represent the true intra-uterine growth retardation (IUGR) among average mothers. *NB_RISK*_*j *_represents a potential neighbourhood-level risk. At the neighbourhood level, the derived potential risk variables described in Table 1 and the DA-level household income variable obtained from census 2006 were fitted into this model separately one at a time as the neighbourhood-level risk (*NB_RISK*_*j*_) to test their associations with LBW, or IUGR in particular. The analysis results are listed in Table 3.

**Table 3:** Parameter estimates of the multi-level analysis of LBW births (Bold rows represent statistically significant results at the 5% level)

CCHS Variable	Estimate	Standard Error	t Value	P > |T|
**Household income by kriging (per $1000)**	**-0.0087**	**0.0026**	**-3.31**	**0.0009**

**Household income by Census (per $1000)**	**-0.0046**	**0.0011**	**-3.99**	**<.0001**

**Self-perceived health (SPH)**	**0.3170**	**0.1229**	**2.58**	**0.0099**

Chronic health conditions (CHC)	0.4143	0.4600	0.90	0.3678

Self-perceived unmet health need (SPUH)	0.7993	0.6334	1.26	0.2070

**Self-perceived stress (SPS)**	**0.6062**	**0.2535**	**2.39**	**0.0168**

Sense of not belonging to local communities (SBC)	0.3009	0.1799	1.67	0.0944

Emotional unhappiness (EU)	2.6028	1.6057	1.62	0.1050

**Food insecurity (FI)**	**1.2511**	**0.4424**	**2.83**	**0.0047**

**Insufficient vegetable intakes (IV)**	**1.8912**	**0.4351**	**4.35**	**<.0001**

Daily smoking (DS)	0.03591	0.02922	1.23	0.2191

Smoking inside home (SIH)	-0.4767	0.7782	-0.61	0.5401

**Physical inactiveness (PI)**	**0.8791**	**0.3502**	**2.51**	**0.0121**

Regular drinking (RD)	-0.8324	0.4418	-1.88	0.0596

Hard drinking (HD)	-0.4907	0.5052	-0.97	0.3314

The estimated parameter values for the two household income variables from kriging and census data are compared for the extent to which their modeled effects explain LBW. As shown in the first two rows in Table 3, these two variables have similar predicting power in statistical modeling to explain their impacts on LBW. Although the parameter for the kriging derived variable is a little lower and the confidence range is a little higher, the difference is small and acceptable. In addition, since the kriging derived variables tend to produce smoother results in predicting the real values, some of the peak values may be masked in the derived neighbourhood-level variables. This may reduce the predicting power for some variables to reveal true associations in terms of statistical significance. For example, both the Sense of Belonging to local Community (SBC) and Regular Drinking (RD) variables show expected associations with LBW, but they are just barely significant at the 5% level. If true values for these two variables can be obtained, greater significance may result. These results suggest future investigation directions to distinguish whether the insignificance is a consequence of the absence of true values or just the lack of epidemiological plausibility. On the other hand, once the associations are established based on the predicted variables, they should represent real statistical associations.

The above multi-level analysis also established significant associations between IUGR and neighbourhood-level self-perceived health conditions (SPH), self-perceived stress (SPS), average physical inactivity (PI), food insecurity (FI) and insufficient vegetable intakes (IF). These associations suggest different aspects of social determinants of LBW, including neighbourhood living conditions that affect the health of mothers, psycho-social factors or social capital of neighbourhoods that put stresses on local residents, local food supply conditions that affect the nutrition of the mothers, and urban design factors that affect physical activity. All of these provide interesting anchors for future analysis. However, since SPS is not spatially autocorrelated, the derived neighbourhood level SPS may only represent an average characteristic of individual members at the neighbourhood level, rather than collective neighbourhood characteristics, such as neighbourhood stressors. The revealed association based on this variable may therefore only represent some connections between IUGR and individual SPS. The interpretation of this association should be made with caution.

## Discussion and Conclusions

Since the CCHS surveys are designed for health studies at the health region level, their direct use at a small-area level is sometimes questionable. However, because of the rich information provided by the data, population health research can be greatly benefited if the data can be used effectively. A spatial autocorrelation test using Moran's I suggested that global spatial dependencies exist for most of the CCHS variables. This satisfies the basic assumption of spatial interpolation methods and makes it possible to use this method to generate health-related indicators at the small-area level.

Based on the cross-validation results and the comparison with census data, kriging provides a good interpolating strategy in comparison with the IDW method for constructing neighbourhood level CCHS variables. The derived CCHS variables at the neighbourhood level are reliable for most of the CCHS variables. However, they may be somewhat smoother than the real values due to the application of a global rule in spatial interpolation. As neighbourhood-level exposures in spatial statistical modeling, the derived neighbourhood characteristics are suitable for exploring potential associations. In other words, once an association between the derived variables and health outcomes is established by statistical testing, it should represent a "real" statistical association. However, since the variation of the interpolated variables is lower than the actual values due to the smoothing effect, they may lose some power in testing the significance of a potential association, especially for an association that is barely significant. Overall, the interpolated values are reasonably well for statistical modeling in public health research at the small-area level. Some interpolated variables, such as SIH, SPS, and HD, may not be so appropriate due to the lack of spatial dependency in the observed data. They should be used with caution.

The other benefit of kriging is the production of standard errors for predicted variables (Figure 8). It can be observed from Figure 8 that the average standard error for household income interpolation is very high. This means that there is considerable uncertainty in the interpolated values. However, since the CCHS is an ongoing survey, data samples can be continually accumulated to produce more reliable interpolation results. The uncertainty is expected to be reduced by involving more cycles in the interpolation process.

It can be observed in Figure 8 that for locations with very few samples, the standard errors are higher and the confidence intervals are larger. This result can be used directly in statistical modeling, such as Bayesian spatial hierarchical modeling, to handle data uncertainty and improve further the reliability of analytical results. For example, the above multi-level model can be similarly constructed using Bayesian hierarchical models with the neighbourhood level model modified as:

$$\begin{array}{l} \text{Level 2}\left(\text{neighbourhood}\right):\\ \beta_{0j}=\gamma_{00}+\gamma_{01}NB\_VAR_{j}+u_{0j}\\ NB\_VAR_{j}∼normal\left(NB\_RISK_{j},NB\_VARANCE_{j}\right). \end{array}$$

Instead of using neighbourhood level risk factors (*NB_RISK*_*j*_) as data, we can consider them as variables with a certain distribution (such as a normal distribution) and use the kriging standard errors to calculate the variances (*NB_VARANCE*_*j*_). Data uncertainty issues can then be effectively handled and the analysis results will be more reliable. In addition to the stochastic standard errors obtained from kriging interpolation, an alternative for the production of standard errors in neighbourhood level predictors is the use of small area methods which take account of spatial dependence [27].

The correlation of socio-economic status and health also makes it appealing to use other spatial interpolation techniques, such as co-kriging, to further improve the spatial interpolation results. Instead of using just the auto-correlated variable of interest for spatial interpolation, co-kriging further makes use of information from other cross-correlated variables to make better predictions. Finding several variables highly cross-correlated to the variable of interest may improve prediction results through co-kriging.

Since the spatial locations of samples are crucial for analyzing spatial dependency and conducting spatial interpolation, the inaccuracy of geocoded locations using postal codes may affect the construction of the semi-varigram and consequently affect interpolated results, especially for rural areas. As explained earlier, geocoding samples with the same postal code to the same location may result in a large nugget value for the constructed semi-variance model. Consequently, weights for small distance samples may be underestimated and greater variance may result. Since the interpolated surfaces (Figures 3 and 4) are not directly used for statistical inference, the aggregation of grid point values from this surface to the small-area level will minimize the above influence caused by the inaccuracy of geocoding using postal code. The accuracy of georeferencing by postal codes is constrained by the precision of postal codes. The uncertainties may not be greatly improved by statistical means. However, if more accurate geographical identifiers, such as street addresses of the sample units can be obtained from the surveys, sample locations will be geocoded much more correctly and small scale geographical studies of health will be greatly benefited.

In addition, if samples are selected more evenly among small areas, such as DAs, the interpolated results will also be improved based on evenly distributed samples. This may be achieved by adjusting the first-stage of the area frame in the sampling design. It is currently done by randomly selecting small areas for sampling. Some of the small areas are therefore not selected, even within several cycles. Theoretically, the design may be improved by selecting all the small areas in the first stage and then randomly select samples within each small area in proportion to its population size. If this is not feasible in one cycle, it might be possible in several cycles to cover all small areas exhaustively. Through a combination of data from several cycles, a set of samples that are suitable for small-area analyses may be obtained.

Increasingly available health survey data bring us not only opportunities, but also challenges in the effective use of these data for public health research. The interpolation procedures in this paper provide an approach to help make use of these data in smaller spatial scales than the designed spatial unit in secondary analysis. While the identified parameters have been chosen for interpolating CCHS data, the general procedures are applicable in general to health-related survey data to obtain neighbourhood characteristics.

## Competing interests

The authors declare that they have no competing interests.

## Authors' contributions

GM carried out to data analysis and paper writing. JL and MET provided advices in the design of the study, gave constructive comments to improve the paper, and made significant revisions to the text of the paper. All authors read and approved the final manuscript.
